# On the Adjacency Matrix of RyR2 Cluster Structures

**DOI:** 10.1371/journal.pcbi.1004521

**Published:** 2015-11-06

**Authors:** Mark A. Walker, Tobias Kohl, Stephan E. Lehnart, Joseph L. Greenstein, W. J. Lederer, Raimond L. Winslow

**Affiliations:** 1 Institute for Computational Medicine, Department of Biomedical Engineering, Johns Hopkins University, Baltimore, Maryland, United States of America; 2 Heart Research Center Göttingen, Clinic of Cardiology and Pulmonology, University Medical Center Göttingen, Göttingen, Germany; 3 German Center for Cardiovascular Research site Göttingen, Germany; 4 Center for Biomedical Engineering and Technology, University of Maryland School of Medicine, Baltimore, Maryland, United States of America; University of Virginia, UNITED STATES

## Abstract

In the heart, electrical stimulation of cardiac myocytes increases the open probability of sarcolemmal voltage-sensitive Ca^2+^ channels and flux of Ca^2+^ into the cells. This increases Ca^2+^ binding to ligand-gated channels known as ryanodine receptors (RyR2). Their openings cause cell-wide release of Ca^2+^, which in turn causes muscle contraction and the generation of the mechanical force required to pump blood. In resting myocytes, RyR2s can also open spontaneously giving rise to spatially-confined Ca^2+^ release events known as “sparks.” RyR2s are organized in a lattice to form clusters in the junctional sarcoplasmic reticulum membrane. Our recent work has shown that the spatial arrangement of RyR2s within clusters strongly influences the frequency of Ca^2+^ sparks. We showed that the probability of a Ca^2+^ spark occurring when a single RyR2 in the cluster opens spontaneously can be predicted from the precise spatial arrangements of the RyR2s. Thus, “function” follows from “structure.” This probability is related to the maximum eigenvalue (*λ*_1_) of the adjacency matrix of the RyR2 cluster lattice. In this work, we develop a theoretical framework for understanding this relationship. We present a stochastic contact network model of the Ca^2+^ spark initiation process. We show that *λ*_1_ determines a stability threshold for the formation of Ca^2+^ sparks in terms of the RyR2 gating transition rates. We recapitulate these results by applying the model to realistic RyR2 cluster structures informed by super-resolution stimulated emission depletion (STED) microscopy. Eigendecomposition of the linearized mean-field contact network model reveals functional subdomains within RyR2 clusters with distinct sensitivities to Ca^2+^. This work provides novel perspectives on the cardiac Ca^2+^ release process and a general method for inferring the functional properties of transmembrane receptor clusters from their structure.

## Author Summary

Many transmembrane receptors have been shown to aggregate into supramolecular clusters that enhance sensitivity to external stimuli in a variety of cell types. Advances in super-resolution microscopy have enabled researchers to study these structures with sufficient detail to distinguish the precise locations of individual receptors. In the heart, efforts have been successful in imaging calcium release channels, which are found in clusters of up to ∼ 100 in the sarcoplasmic reticulum membrane of cardiac myocytes. We showed in a recent study how the precise cluster structure affects the frequency of spontaneous release events known as calcium “sparks.” Here we have developed an analytical model of calcium spark initiation that clearly illustrates how the structure controls spark likelihood. We then applied this model to a collection of channel cluster structures obtained using super-resolution microscopy, revealing spatial gradients in the functional properties of individual channels. This work provides insight into the calcium release process in the heart and a framework for evaluating functional heterogeneity in populations of receptor clusters using structural information alone.

## Introduction

**Fig 1:**
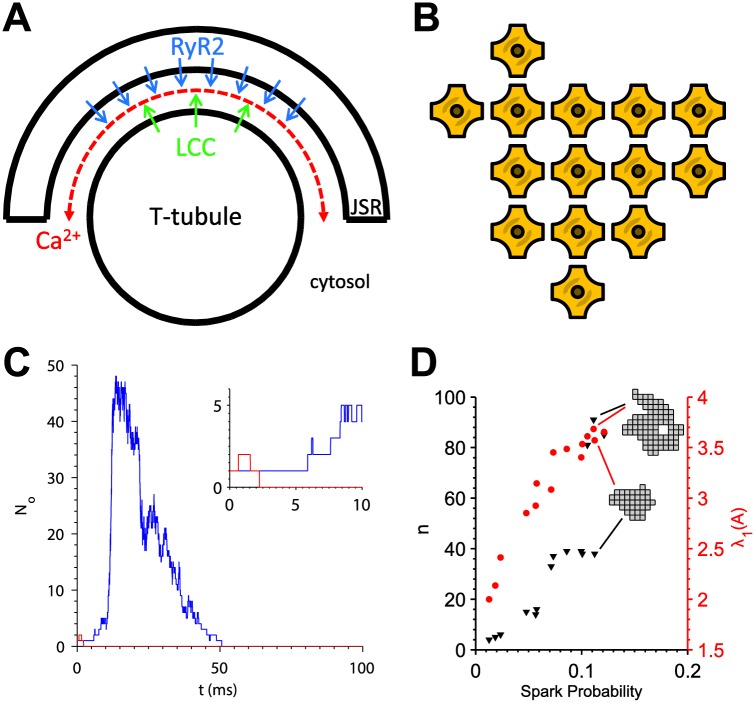
The Ca^2+^ sparks are stochastic events that occur at release sites in the cardiac myocyte. (A) Cross-section of release site structure. Deep invaginations in the cell membrane known as transverse tubules meet with the JSR to form a narrow subspace containing a cluster of RyR2s (*blue*), which release Ca^2+^ inside the cell. Arrows indicate direction of Ca^2+^ flux. (B) Example cluster of RyR2s forming a packed lattice. (C) Representative simulations from the 3D spark model showing the number of open channels (*N*_*O*_) out of 49. After a single channel is opened at *t* = 0, it probabilistically succeeds (*blue*) or fails (*red*) to trigger a spark. Inset shows the spark initiation phase. (D) Relationship of cluster size (*n*, *triangles*) and maximum eigenvalue (*λ*_1_, *circles*) with Ca^2+^ spark probability in the 3D spark model [4]. Two embedded cluster lattices are shown to illustrate that RyR2 clusters can exhibit equal Ca^2+^ spark probability despite differences in the number of channels due to differences in shape.

Mechanical contraction of the heart occurs as a result of intracellular Ca^2+^ release in cardiac myocytes. L-type Ca^2+^ channels (LCCs) and a packed cluster of up to 100 Ca^2+^-sensitive Ca^2+^-release channels [1, 2], known as ryanodine receptors (RyR2s), are co-located at discrete subcellular junctions within the cell (Fig 1A). These Ca^2+^ release units (CRUs) are formed by deep invaginations of the cell membrane containing LCCs, known as transverse-tubules (TTs), and the junctional sarcoplasmic reticulum (JSR) membrane, a cisternal sheet containing the RyR2s that wraps around the TT to form a narrow subspace ∼ 15 nm in width. During excitation-contraction coupling (ECC), electrical stimulation increases the probability of LCC openings and influx of Ca^2+^ into these subspaces. Binding of Ca^2+^ to the closely-apposed RyR2s [3] increases their open probability and release of Ca^2+^ from JSR stores in a process known as Ca^2+^-induced Ca^2+^ release (CICR). Further Ca^2+^ release from RyR2s activates surrounding RyR2s via a local rise in subspace Ca^2+^ concentration. Understanding this process is critical to our understanding of cardiac physiology in health and disease.

Isolated release events known as Ca^2+^ “sparks” underlie the cell-wide release of Ca^2+^ that occurs on every heartbeat when the RyR2s are activated by the opening of voltage-sensitive Ca^2+^ channels. Ca^2+^ sparks are also observed in resting myocytes when initiated by the spontaneous opening of a single RyR2 that then probabilistically triggers Ca^2+^ release from the rest of the cluster. Here, the probability that an RyR2 is in an open state at any point in time will be referred to as its open probability, and the probability that a sufficiently large percentage of the RyR2s open for a spark to occur will be referred to as spark probability (*p*_*S*_).

Spark probability is an important physiological parameter that in part controls the frequency of sparks [4, 5]. There is significant experimental evidence that not all junctional RyR2 openings result in Ca^2+^ sparks [6–9]. These non-spark openings may in part be attributed to non-junctional RyR2s located outside the release site [10, 11]. However, mathematical modeling suggests that junctional RyR2 openings can fail to trigger Ca^2+^ sparks and are sufficient to account for the non-spark openings [4, 5, 12, 13]. In support of this, recent experiments using Ca^2+^ nanosensors targeted to the release site have demonstrated locally elevated Ca^2+^ concentration in the subspace due to spontaneous junctional RyR2 openings [14, 15]. This is also consistent with the observation that the majority of Ca^2+^ released via non-spark openings is extruded through the Na^+^/Ca^2+^ exchanger, which is localized near the junctions [15]. Therefore there is compelling evidence suggesting that Ca^2+^ spark initiation is likely a probabilistic process.

Many studies have implicated Ca^2+^ sparks in heart disease. For example, spark frequency is increased in heart failure [7, 16], which is associated with decreased JSR Ca^2+^ content and thus impaired contractile function [17]. Ca^2+^ sparks may also cause spontaneous Ca^2+^ waves [18, 19] that promote cellular arrhythmias [20]. Heterogeneity in the Ca^2+^ sensitivity of RyR2 clusters has also been implicated in the occurrence of arrhythmic Ca^2+^ alternans [21]. Therefore factors that influence Ca^2+^ spark probability are likely to be involved in mechanisms driving pathological cardiac dysfunction.

Advancements in super-resolution imaging techniques have enabled the study of nanoscale receptor organization in a variety of cell types [22, 23]. In cardiac myocytes, stimulated emission depletion (STED) microscopy has been applied to study TT remodeling in heart failure at nanometer resolution [24]. Within the cardiac Ca^2+^ release sites, RyR2s are known to form tightly-packed clusters from electron microscopy studies *in vivo* [1] (Fig 1B), which is supported by the observation that the channels organize into packed lattices with ∼ 31 nm spacing *in vitro* [25]. Super-resolution imaging studies of RyR2 clusters using super-resolution microscopy in cardiac myocytes have revealed that they are heterogeneous in size and shape [2].

We recently developed a three-dimensional, biophysically-detailed model of cardiac Ca^2+^ release events, which we will refer to as the 3D spark model. Fig 1C shows example simulations, in which a single open channel either fails or succeeds to activate the remaining RyR2s. We obtained realistic RyR2 clusters obtained using STED microscopy and showed that the precise spatial arrangement of RyR2s critically influences spark probability [4]. Larger, more compact clusters exhibited higher spark probability than smaller, fragmented ones. Representing RyR2 clusters as a two-dimensional lattice, we found that the maximum eigenvalue (*λ*_1_) of the lattice’s adjacency matrix predicts the Ca^2+^ spark probability of the cluster. Properties of the eigenvalues and eigenvectors of a graph’s adjacency matrix have been widely studied, and *λ*_1_ in particular is known to be a measure of the interconnectedness of the graph [26, 27]. *λ*_1_ was found to be a more accurate predictor of spark probability than is the total number of channels (*n*), which does not consider structural aspects of the cluster. Fig 1D shows the relationship between spark probability, *n*, and *λ*_1_ for two RyR2 clusters analyzed previously [4]. The two embedded cluster lattices have nearly equal spark probability, despite one being much larger than the other. This is because the larger lattice contained four empty spaces in the interior. However, the *λ*_1_ values were similar for these two clusters and therefore consistently correlated with spark probability.

Here we present an analytical model of the Ca^2+^ release process and derive the relationship between *λ*_1_ and spark probability. The model is applied to realistic RyR2 clusters obtained using stimulated emission depletion (STED) microscopy. We found through an eigendecomposition that some RyR2 clusters possess functional subdomains with distinct sensitivity to Ca^2+^. This work outlines a unique approach to understanding CICR and provides a theoretical framework for comparing the physiological function of protein clusters based solely on structural information.

## Results

### Contact network model

**Fig 2:**
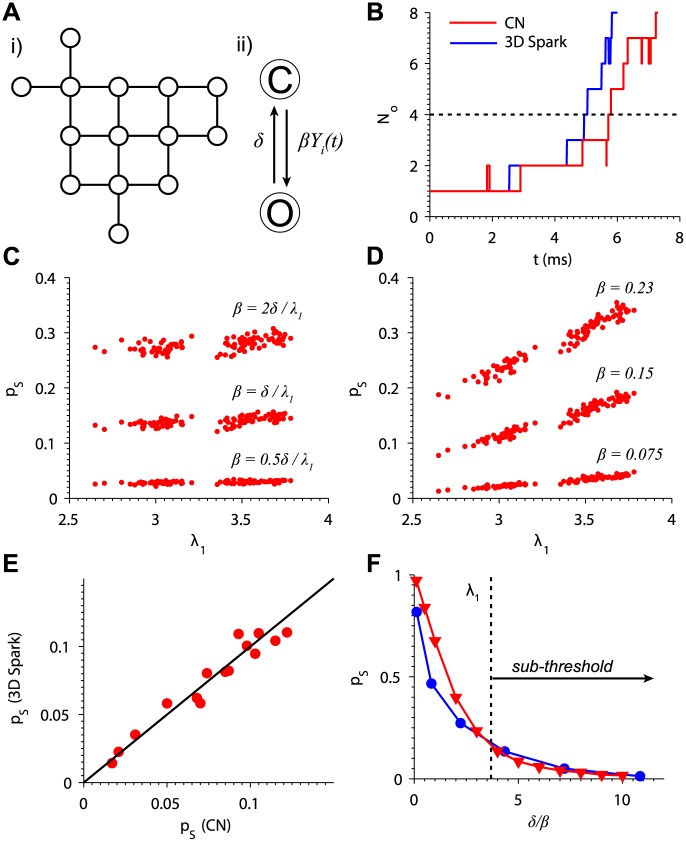
The contact network model reproduces RyR2 channel gating during Ca^2+^ spark initiation. (A) Contact network model schematic showing (i) a diagram of the structure of the RyR2 lattice from Fig 1 (B) with lines connecting adjacent channels and (ii) the Markov model representing each channel with closed (C) and open (O) states. The opening rate of each channel *i* scales with the number of open adjacent channels *Y*_*i*_(*t*). (B) Example simulations showing CN model (*red*) and 3D spark model (*blue*) RyR2 gating behavior during the spark initiation phase. Spark generation is considered successful if *N*_*O*_ ≥ 4 (dashed line). (C) Spark probabilities for a collection of 107 STED-informed RyR2 clusters for threshold (middle), sub-threshold (lower), and supra-threshold (upper) values of *β*. (D) Spark probabilities when the value of *β* was set to constant values across all clusters. (E) *β* was adjusted to the nominal value of 0.115ms^−1^ to maximize the correlation with spark probabilities from the 3D spark model for a collection of 15 representative clusters (*R* ^2^ = 0.939). (F) Spark probability (*p*_*S*_) of a 7 × 7 lattice of RyR2s as a function of *δ*/*β* in the CN (*red*) and 3D spark (*blue*) models (see text for details). The CN model is in the sub-threshold regime when *δ*/*β* is to the right of *λ*_1_ (*black dotted line*).

In this section, we present results using a contact network (CN) model of RyR2 cluster activation where channels are coupled through local interactions with their neighbors. Contact network models are widely used to study the spread of disease due to contact between infected and susceptible individuals [28]. In our model, interactions instead arise from Ca^2+^-dependent activation due to local influx and diffusion of Ca^2+^, which causes neighboring channels to open. For simplicity, we assume that the local Ca^2+^ concentration gradient near an open RyR2 declines rapidly enough in space such that only adjacent RyR2s interact [4, 29, 30]. Each channel transitions stochastically between open and closed states (Fig 2A). If an RyR2 channel *i* has *Y*_*i*_(*t*) neighboring RyR2 channels that are open, its opening rate is *βY*_*i*_(*t*), where *β* is a constant parameter. Therefore *β* is the RyR2 opening rate when one nearest neighbor RyR2 is open. Note that in the full biophysical 3D spark model, the RyR2 opening rate when all neighbors are closed is very small (∼ 9 × 10^−7^ms^−1^). Therefore we have taken this rate to be zero in this formulation. The value of *β* is varied in our analyses. The RyR2 closing rate, *δ*, is assumed to be a constant 0.5ms^−1^. Derivation of the model and parameters are given in Methods and Models.

The CN model is able to capture RyR2 gating dynamics during the initiation phase of Ca^2+^ sparks. We used the Stochastic Simulation Algorithm of Gillespie [31] to simulate the stochastic CN model. Fig 2B shows traces of the number of open channels (*N*_*O*_) during representative simulations of spark initiation in the 3D spark and CN models for a 7 × 7 lattice cluster. A single RyR2 is opened at *t* = 0, which then triggers openings of other channels. The CN model qualitatively reproduces channel gating behavior during the initiation of the spark. In the 3D spark model, Ca^2+^ sparks occur with greater than 95% probability if a minimum of four channels open. Therefore, we define this as the minimum number for successful spark initiation in both models. We also assume that each RyR2 in the cluster is equally likely to open spontaneously, and so the first open channel is chosen at random.

The advantage of developing the CN model is that we can derive analytical relationships between the dominant eigenvalue of the RyR2 lattice’s adjacency matrix, *λ*_1_, and spark probability. We show (see Eq (9) in Methods and Models) that for a deterministic mean-field approximation of the model, RyR2 open probability decays to zero when

$$\begin{array}{c} \lambda_{1}<\frac{\delta }{\beta }. \end{array}$$ (1)

This implies that, in the mean-field approximation, *δ*/*β* is a stability threshold for *λ*_1_ at which RyR2 activity switches from decay to growth. While it was not immediately clear how this threshold related to the behavior of the full stochastic CN model, we expected that the model would exhibit constant spark probability when *λ*_1_ = *δ*/*β*. That is, for a set of cluster structures each with a different value of *λ*_1_, the spark probability would be consistent across clusters when each cluster’s opening rate was set to *β* = *δ*/*λ*_1_. Fig 2C shows the spark probability for a collection of 107 RyR2 clusters obtained using STED microscopy (see [4] for imaging methods). For each simulation, *λ*_1_ was computed for the cluster and *β* was set to the threshold value *δ*/*λ*_1_. The range of spark probabilities across all clusters was narrow (0.14±0.0078). This was also observed when using sub-threshold values *β* = 0.5*δ*/*λ*_1_ (0.029±0.0024) and a supra-threshold values *β* = 2*δ*/*λ*_1_ (0.28±0.012). Therefore spark probability is constant when *β* is scaled inversely with *λ*_1_. For comparison, we also plotted spark probability when *β* is set to a single value across all clusters (Fig 2D). In this case, spark probability increased with *λ*_1_ in agreement with the 3D spark model (see Fig 1D).

The CN model was able to accurately predict Ca^2+^ spark probability for a range of cluster geometries. We estimated the spark probabilities for a collection of 15 RyR2 clusters obtained using STED microscopy. The value of *β* was adjusted until the spark probabilities in the CN model correlated with those of the 3D spark model (Fig 2E). Maximal correlation was achieved for *β* = 0.115 (*R* ^2^ = 0.939), which gives the value of *δ*/*β* = 4.35. Note that the theoretical value of *λ*_1_ for any cluster is bounded above by the maximum number of channel neighbors (4) [26]. Consequently, *δ*/*β* = 4.35 > *λ*_1_ implies that the system is always sub-threshold for any cluster structure under normal physiological conditions.

The CN model also predicts spark probability for different opening rates. To show this, we first estimated *p*_*S*_ in the 3D spark model for a 7 × 7 cluster with the opening rate scaled by a constant factor. We then scaled *β* = 0.115 by the same factor and determined *p*_*S*_ in the CN model. This was repeated for a range of scaling factors. Noting that the closing rates *δ* are the same in both models, we could directly compare *p*_*S*_ in the two models by plotting it as a function of *δ*/*β*, where *β* is the scaled value. For the 3D spark model, *β* is the value used in the corresponding CN simulation. In both models, spark probability fell rapidly as *δ*/*β* approached *λ*_1_ from the left before decreasing gradually to the right of *λ*_1_. This suggests that spark probability is more sensitive to RyR2 gating kinetics when the opening rate is elevated. From the data in this section, we concluded that the CN model is able to accurately predict *p*_*S*_ over a range of opening rates and cluster geometries.

### Ca^2+^ diffusion in the CN model

Cardiac Ca^2+^ release is actively regulated under normal conditions and modulated in various diseases. To study this regulation, we expanded the CN model by deriving a simple model of Ca^2+^ diffusion between RyR2 Ca^2+^ sources. The parameter *β* was estimated using this diffusion model and a model of RyR2 gating. All parameters were taken from Walker et al. [4], except for the effective Ca^2+^ diffusion coefficient (*d*_*C*_), which was adjusted to give *β* = 0.115 as determined in the previous section.

**Fig 3:**
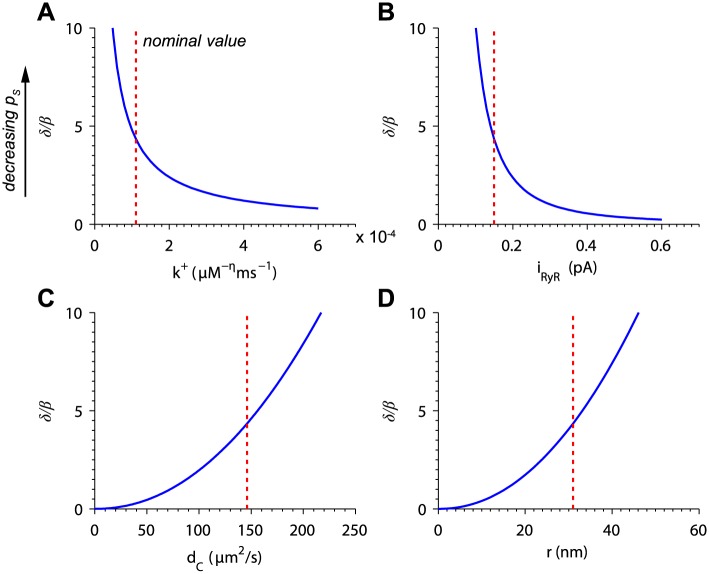
Dependence of *δ*/*β* on biophysical properties of the cardiac Ca^2+^ release site. Dependence on (A) the RyR2 opening rate constant, (B) unitary RyR2 current, (C) effective Ca^2+^ diffusion coefficient, and (D) distance between channel pore and neighboring Ca^2+^ binding site. Red dotted lines indicate nominal parameter values from Walker et al. [4], except for *d*_*C*_, which was adjusted to give *β* = 0.115 as determined in Fig 2E.

A number of signaling molecules regulate RyR2 channels, affecting their opening rate. This includes RyR2 phosphorylation by Ca^2+^/calmodulin-dependent protein kinase II (CaMKII) and protein kinase A (PKA) [32, 33] and JSR Ca^2+^ concentration [34]. Channel gating can also be altered under oxidative stress [35] and by genetic mutations [36, 37]. As shown in Fig 3A, *δ*/*β* is inversely proportional to the channel opening rate constant (*k* ^+^), reflecting the increased Ca^2+^ spark frequency observed under such conditions [7, 38, 39]. Note that the closing rate is *δ* and therefore scales *δ*/*β* linearly. Increasing the unitary channel current (*i*_*RyR*_) resulted in a decrease in *δ*/*β* (Fig 3B). This behavior is consistent with experimental evidence [40], in which decreased *i*_*RyR*_ resulted in lowered spark frequency.

The CN model was also sensitive to parameters affecting the diffusion of Ca^2+^ ions in the release site subspace. Fig 3C shows the dependence of *δ*/*β* on *d*_*C*_. As *d*_*C*_ increases, Ca^2+^ ions are more likely to escape the nanodomain around the open channel, thus decreasing spark probability. Uniformly increasing the distance between the open channel pore and neighboring Ca^2+^ binding site increased *δ*/*β* so as to decrease spark probability (Fig 3D).

In this section, we have used a simple diffusion model to probe the effects of perturbations to biophysical properties of the release site including the opening rate, unitary channel current, Ca^2+^ diffusion coefficient, and inter-channel spacing. The CN model suggests that minor modifications to these parameters can alter the stability of the system, thus leading to significant changes in spark probability.

### Linear mean-field CN model

Up to this point, we have considered spark probability when each RyR2 is equally likely to open first. An emergent property of the 3D spark model was that the probability of a spark occurring varied with the choice of initiating RyR2 [4]. Channels closer to the epicenter of the cluster were more likely to trigger sparks because they have more possible combinations of first, second, third, etc. neighbors along which channel openings could propagate. Likewise, channels on the periphery of the cluster were less likely to trigger sparks.

**Fig 4:**
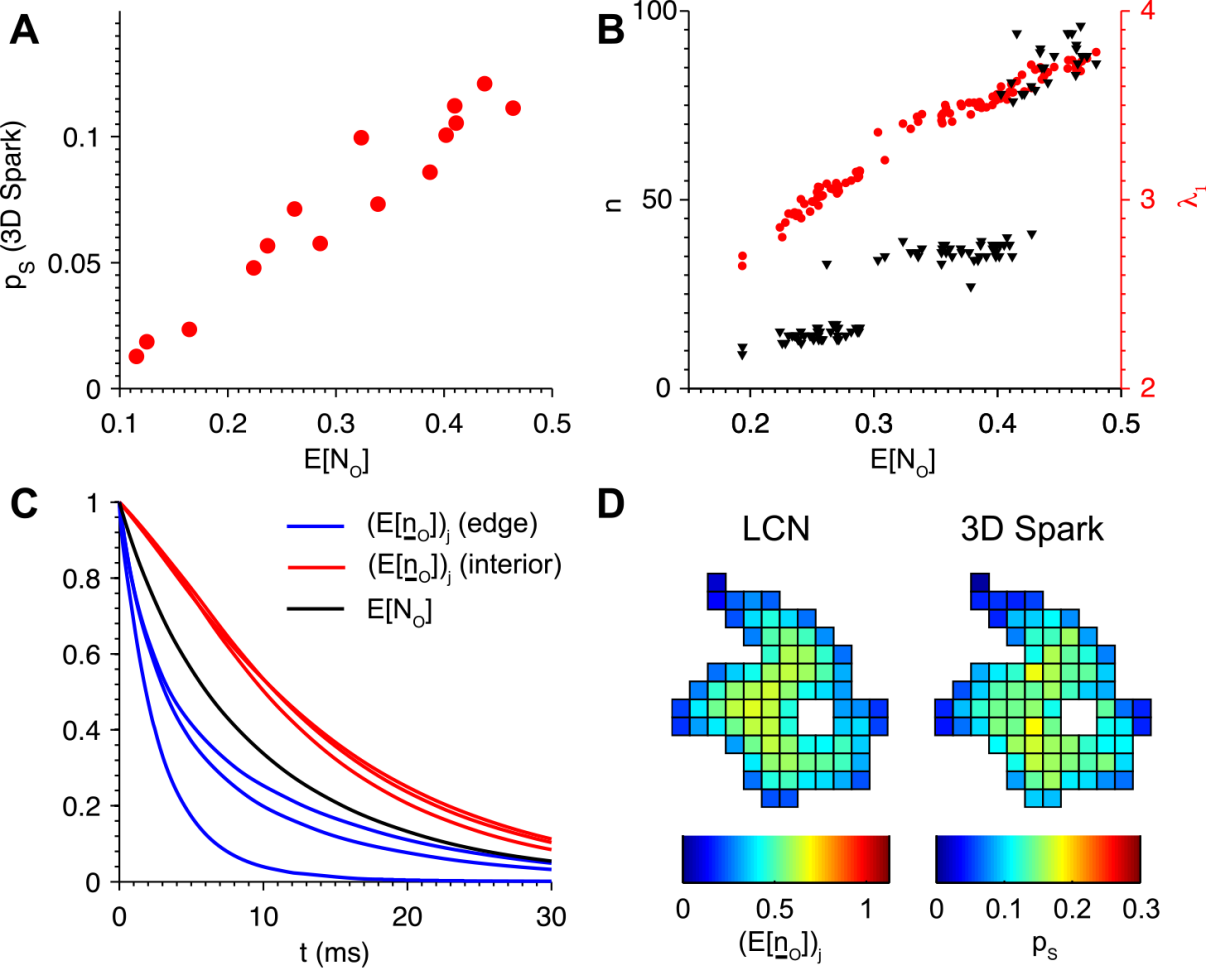
The expected number of open channels in the linearized mean-field CN (LCN) model predicts spark probability. (A) Expected number of open channels (E[*N*_*O*_]) of the LCN model at *t* = 8 ms strongly correlated with *p*_*S*_ of the 3D spark model (*R* ^2^ = 0.934). Data shown for a collection of 15 clusters. (B) E[*N*_*O*_] also correlated with *λ*_1_ (*red*), but not with the number of channels in the cluster (*n*, *black*). Data points are from collection of 107 clusters. (C) Time dependence of the expected number of open channels for different initiating RyR2s ($\text{E}\left[{\underline{n}}_{O}\right]$) on edge of the cluster (*blue*), in the interior (*red*), and the average (E[*N*_*O*_], *black*) for the cluster shown in the following panel. (D) Heatmaps of $\text{E}\left[{\underline{n}}_{O}\right]$ (*left*) and *p*_*S*_ estimated using the 3D spark model (*right*) illustrates intra-cluster gradients in spark probability.

We derive a linear mean-field representation of the CN (LCN) model (see Methods and Models) to quantitatively study how spark probability depends on the position of the initiating RyR2. The LCN model can be used to compute the expected number of open channels as a function of time. We reasoned that a greater expected number of open channels during the spark initiation phase would imply that sparks are more likely to occur and therefore would correlate with *p*_*S*_. Using the LCN model, we derived an expression for the expected number of open channels, E[*N*_*O*_] (see Eq (15)), and computed its value for a collection of 15 RyR2 clusters. We find that E[*N*_*O*_] derived in the LCN model correlated with *p*_*S*_ in the 3D spark model (*R* ^2^ = 0.934, Fig 4A). Note that the equation for E[*N*_*O*_] is time-dependent, but the results were not sensitive to our choice of the time point *t* (*R* ^2^ = 0.933 and 0.923 at *t* = 4 and 12 ms, respectively).

To further establish the relationship between E[*N*_*O*_] and *p*_*S*_, we compared E[*N*_*O*_] to *λ*_1_ for a broader collection of 107 clusters obtained from STED microscopy (Fig 4B). A strong correlation between these variables was present, in contrast to the number of channels in the cluster, which was not consistently correlated with E[*N*_*O*_]. Recall that these conclusions were also drawn from the data of Fig 1D for a smaller collection of clusters. Taken together, these data suggest that *λ*_1_ and E[*N*_*O*_] are both accurate predictors of *p*_*S*_, while by itself the number of channels without regard to relative channel locations is not.

The LCN model was used to compute the vector whose elements are the expected number of open channels given each possible initiating RyR2. We denote this vector $\text{E}\left[{\underline{n}}_{O}\right]$ (see Eq (14)), where each element ${\left(\text{E}\left[{\underline{n}}_{O}\right]\right)}_{j}$ is the expected number of open channels given that channel *j* is opened initially. Note that our nominal value of *δ*/*β* is in the sub-threshold regime, which implies that E[*N*_*O*_] and $\text{E}\left[{\underline{n}}_{O}\right]$ both decay in the LCN model (see Eqs (15) and (14)). Fig 4C shows how ${\left(\text{E}\left[{\underline{n}}_{O}\right]\right)}_{j}$ was initially 1, reflecting the first open channel, and decayed in time. This occurred at varying rates within an individual cluster, depending on the choice of initiating RyR2. ${\left(\text{E}\left[{\underline{n}}_{O}\right]\right)}_{j}$ decayed more rapidly for channels *j* near the edge compared to those near the center, consistent with the lower peripheral spark probabilities estimated using the 3D spark model (Fig 4D). This is because channels near the edge have fewer adjacent channels to trigger, and therefore it is less likely that a second channel will open before the first closes. Furthermore, the peripheral channels tend to have fewer second, third, etc. neighbors that can potentially be activated compared to central channels.

We conclude that both the expected number of open channels in the LCN model is strongly correlated with spark probability. This fact will be used to further analyze spatial gradients in spark probability that depend on which RyR2 is opened initially.

### Characterization of functional subdomains by the eigenmodes

The LCN model can be decomposed into a set of independent eigenmodes by taking the similarity transform of the adjacency matrix: **A** = **V** **D** **V** ^*T*^, where **V** is the modal matrix whose columns are formed by a set of orthonormal eigenvectors $\left\{{\underline{v}}_{1},{\underline{v}}_{2},...,{\underline{v}}_{n}\right\}$ and **D** is a diagonal matrix of eigenvalues {*λ*_1_, *λ*_2_, …, *λ*_*n*_} in descending order. Note that **A** is symmetric such that **V** ^−1^ = **V** ^*T*^. The *i* ^th^ eigenmode is defined by the pair $\lambda_{i}-{\underline{v}}_{i}$, in which *λ*_*i*_ determines the rate of decay of the eigenmode in time and the values ${\left({\underline{v}}_{i}\right)}_{j}$ determine the membership of channel *j* in the eigenmode. We derived an expression for $\text{E}\left[{\underline{n}}_{O}\right]$ as a weighted sum of the eigenvectors

$$\begin{array}{c} E[{\underline{n}}_{O}\left(t\right)]=\sum_{i=1}^{n}b_{i}\left(t\right){\underline{v}}_{i}, \end{array}$$ (2)

where the weights $b_{i}(t)=e^{(\beta \lambda_{i}-\delta )t}{\underline{u}}^{T}{\underline{v}}_{i}$, with $\underline{u}$ being the all-one-vector. A similar expression for E[*N*_*O*_] is given by

$$\begin{array}{c} E[N_{O}\left(t\right)]=\sum_{i=1}^{n}c_{i}\left(t\right), \end{array}$$ (3)

where $c_{i}(t)=\frac{1}{n}e^{(\beta \lambda_{i}-\delta )t}{\left({\underline{u}}^{T}{\underline{v}}_{i}\right)}^{2}=\frac{1}{n}b_{i}(t){\underline{u}}^{T}{\underline{v}}_{i}$. Therefore $\text{E}\left[{\underline{n}}_{O}\right]$ and E[*N*_*O*_] are essentially equal to weighted sums of the eigenmodes. The derivation of these equations can be found in Methods and Models.

In the previous section, we presented further evidence of the relationship between *λ*_1_ and spark probability as well as intra-cluster spatial gradients in spark probability. A natural question to then ask is: does the dominant eigenvector (${\underline{v}}_{1}$) corresponding to *λ*_1_ give us information about these gradients? Furthermore, how significant are other eigenmodes?

**Fig 5:**
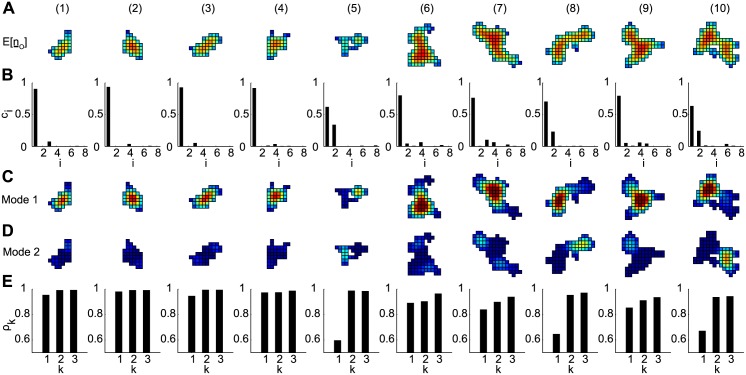
Intra-cluster spatial gradients in spark probability are characterized by one or two eigenmodes. Data are shown for 10 clusters obtained from STED microscopy. (A) Spatial gradients in $\text{E}\left[{\underline{n}}_{O}\right]$. (B) Fractional eigenmode contributions to E[*N*_*O*_] corresponding to the 10 greatest eigenvalues of the adjacency matrix. (C) Dominant eigenmode ($b_{1}{\underline{v}}_{1}$) corresponding to *λ*_1_. (D) Subdominant eigenmode corresponding to the second greatest *b*_*i*_. Negative values shown as 0 for clarity. (E) Correlation coefficients between the values of $\text{E}\left[{\underline{n}}_{O}\right]$ from panel (A) and the (1) dominant eigenmode, (2) sum of dominant and subdominant eigenmodes, and (3) sum of dominant, subdominant, and tertiary eigenmodes. Color scales in all panels are the same as in Fig 4D.

The spatial distribution of $\text{E}\left[{\underline{n}}_{O}\right]$ is shown for collection of 10 RyR2 clusters in Fig 5A. We further defined $c_{i}=c_{i}(\hat{t})/\sum_{j}c_{j}(\hat{t})$ at $\hat{t}=8$ ms, which gives the fractional contribution of the *i* ^th^ eigenmode to $N_{O}(\hat{t})$. We decomposed these clusters into their eigenmodes and plotted the values of *c*_*i*_ corresponding to the 8 greatest eigenvalues in Fig 5A. In most cases (clusters (1)-(4), (6), (7), (9)), *c*_1_ was the only large value, implying that the dominant eigenmode characterized the behavior of the LCN model. Clusters (5), (8), and (10), however, exhibited another significant *c*_*i*_ corresponding to a subdominant eigenmode.

Examining the dominant eigenmode of each cluster, we found that for clusters characterized by only the dominant eigenmode, there was a single locus of elevated membership in the dominant eigenvector corresponding to the channels *j* with greatest values ${\left(\text{E}\left[{\underline{n}}_{O}\right]\right)}_{j}$ (Fig 5C). Furthermore, the eigenmode’s spatial gradients resembled the full solution in Fig 5A. For clusters with a subdominant eigenmode ((5), (8), (10)), however, the dominant eigenvector did not fully characterize the spatial gradients in $\text{E}\left[{\underline{n}}_{O}\right]$. For these clusters, the subdominant eigenmode accounted for areas of high $\text{E}\left[{\underline{n}}_{O}\right]$ that were not included in the dominant eigenmode (Fig 5D). In addition, the subdominant eigenmodes were insignificant in the other clusters.

To quantitatively assess how well the dominant and subdominant eigenmodes characterize spark probability, we computed the correlation coefficients between the $\text{E}\left[{\underline{n}}_{O}\right]$ and the dominant (*k* = 1), the sum of dominant and subdominant (*k* = 2), and sum of the dominant, subdominant, and a tertiary eigenmode corresponding to the third largest *c*_*i*_ (*k* = 3) (Fig 5E). Clusters well-described by the dominant eigenmode generally yielded high *ρ*_1_ > 0.84, indicating that ${\underline{v}}_{1}$ was sufficient to characterize the spatial gradients in spark probability. For the clusters with significant subdominant eigenmodes, *ρ*_1_ was lower (< 0.68), and the second eigenmode was required to establish a correlation. Note that inclusion of the tertiary eigenmode did not greatly improve the correlation, suggesting that the first two eigenmodes were most significant.

In this section, we characterized the intra-cluster spatial gradients in spark probability in terms of the eigenvalues and eigenvectors of the adjacency matrix. In the majority of cases, the dominant eigenmode $\lambda_{1}-{\underline{v}}_{1}$ was sufficient to approximate the gradients. Clusters (5), (8), and (10) of Fig 5, however, possessed secondary subdomains of channels separated from the dominant subdomain by a bottleneck (i.e. dumbbell-like morphology). These functional subdomains generally contained channels with lower spark probability than the dominant subdomain. This is consistent with Eq (2), which indicates that these secondary subdomains are also characterized by a decay rate 1/*λ*_*s*_ > 1/*λ*_1_ and therefore would be expected to have lower spark probability.

### Perturbation analysis

**Fig 6:**
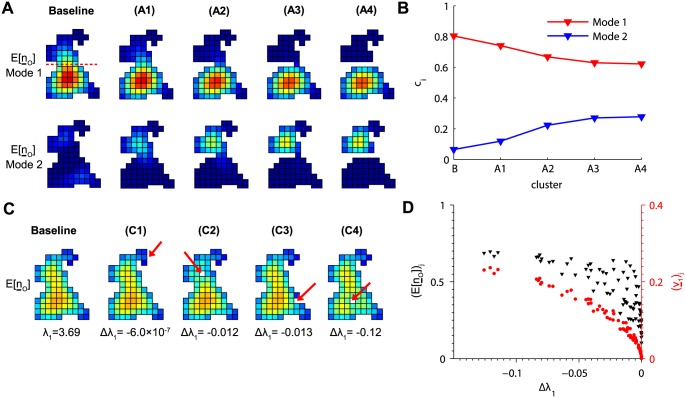
Perturbation analysis of lattice structure. (A) Dominant (*top*) and subdominant (*bottom*) eigenmodes for six variations of cluster (6) in Fig 5. In clusters (A1)-(A4), channels are progressively removed from left to right along the row indicated by the dashed line on the baseline cluster. (B) Values of eigenmode weights *c*_*i*_ as the channels are removed. (C) Removal of a single channel indicated by the arrows caused varying reductions in *λ*_1_ depending on its position. The change in *λ*_1_ compared to the baseline cluster (Δ*λ*_1_) is shown for each cluster (C1)-(C4). Heatmaps show $\text{E}\left[{\underline{n}}_{O}\right]$ in each case. (D) Change in *λ*_1_ when removing a single channel *j* from the baseline cluster. ${\left({\underline{v}}_{1}\right)}_{j}$ is the channel’s corresponding element in the dominant eigenvector (*red*) and ${\left(\text{E}\left[{\underline{n}}_{O}\right]\right)}_{j}$ is its element in $\text{E}\left[{\underline{n}}_{O}\right]$ (*blue*) in the baseline cluster. Δ*λ*_1_ is again the change in *λ*_1_ upon removing channel *j*. Heatmap color scales are the same as in Fig 4D.

It is not clear how one can determine whether a cluster is characterized by a single eigenmode or dominant-subdominant pair of eigenmodes without performing the eigendecomposition computations. For example, comparing clusters (6) and (8) in Fig 5, it is not immediately obvious why (8) requires both modes and (6) does not. To better understand this relationship, we progressively severed the connection between two functional subdomains at the top and bottom of cluster (6). In Fig 6A, we removed channels from this cluster proceeding left to right along the row of channels indicated by the dashed line in the baseline cluster. A subdominant eigenmode emerged as the channels were removed. The dominant eigenmode remained in the lower subdomain, while the subdominant eigenmode formed in the upper region. Note the formation of two disjoint subclusters in cluster (A4), which have eigenmodes similar to when connected by a single channel in (A3). The formation of a secondary subdomain is further demonstrated by an increase in the value of *c*_*i*_ for the subdominant eigenmode (Fig 6B). In this example, the subdominant eigenmode appeared after removing only one channel and gradually became more prominent with the removal of additional channels. Therefore, the formation of a subdominant eigenmode can be quite responsive to reductions in the region dividing two possible subdomains, each distinguished by different propensities for sparks.

We next investigated how sensitive spark probability is to small changes in lattice shape. Fig 6C shows a series of clusters in which only a single channel was removed from the original cluster. As expected, removing a channel along the upper edge as in cluster (C1) where spark probability is low resulted in a small change in *λ*_1_ (Δ*λ*_1_). Discarding a central channel as in cluster (C4) resulted in the greatest change. One may expect that removing channels with the higher spark probability would cause a greater decrease in *λ*_1_. However, the channel removed in cluster (C2) corresponded to a greater element in $\text{E}\left[{\underline{n}}_{O}\right]$ than the channel in (C3), yet the change in *λ*_1_ was less. This is because the channel in cluster (C3) had greater membership in the dominant eigenmode. To illustrate this, we systematically removed each channel one at a time from the baseline cluster and calculated Δ*λ*_1_. Fig 6D shows that there was a consistent relationship between Δ*λ*_1_ and the discarded channel *j*’s corresponding element of the dominant eigenvector ${\left({\underline{v}}_{1}\right)}_{j}$ in the original cluster but not ${\left(\text{E}\left[{\underline{n}}_{O}\right]\right)}_{j}$. Therefore element *j* of the dominant eigenvector determines the extent by which spark probability decreases when a single channel *j* is removed.

## Discussion

We have shown in previous work that the precise structure of RyR2 channel clusters influences properties of Ca^2+^ release [4]. In particular, the probability of a Ca^2+^ spark occurring when an RyR2 opens spontaneously depends strongly on the arrangement of the RyR2s in the subspace. This has implications for Ca^2+^ cycling in the cell, as Ca^2+^ spark probability controls the frequency of Ca^2+^ sparks and the excitability of the cluster [5]. An emergent property of this biophysically-detailed model was that *λ*_1_ is a strong predictor of Ca^2+^ spark probability.

Here we presented a model similar to those used to study the spread of contagion, such as in disease epidemics [41]. In this model, a single RyR2 is opened initially, which increases the open probability of its neighbors via a local rise in Ca^2+^ concentration. After deriving a linearized mean-field formulation of the system, we showed that the open probability of the RyR2s is extinguished when *λ*_1_ < *δ*/*β*. Therefore *λ*_1_ governs a stability threshold for spark generation. In the stochastic model, spark probability was constant across all clusters when *λ*_1_ = *δ*/*β*. Therefore, if one compares any two different RyR2 clusters, the cluster with lower *λ*_1_ value would need a lower *δ*/*β* ratio (i.e. greater RyR2 mean open time or opening rate) to achieve the same spark probability as the other cluster. This explains why *λ*_1_ is correlated with Ca^2+^ spark probability. Cator and Van Mieghem derived a second-order CN model, with which they showed that the true threshold for the system to exhibit exponentially long transients is in fact bounded from above by *λ*_1_ [42]. Nevertheless, the first-order model presented here was sufficient to account for the relationship between *λ*_1_ and spark probability.

It is known that the maximum eigenvalue of a graph’s adjacency matrix is related to the number of walks on the graph [26]. Specifically, if *W*_*k*_ is the number of possible walks of length *k* on a graph with *n* vertices, then $W_{k}\approx n\lambda_{1}^{k}$ when *k* is large. Furthermore, *W*_*k*_ is proportional to ${\left({\underline{u}}^{T}{\underline{v}}_{1}\right)}^{2}$ when *k* is large. Recall that this term also appears in the expression for *c*_1_. It is no coincidence that *W*_*k*_ and E[*N*_*O*_] are related. Intuitively, a greater number of walks implies that there are more possible contiguous sets of RyR2s along which channel openings can propagate. This is essentially the underlying relationship between *λ*_1_ and Ca^2+^ spark probability.

An eigendecomposition of the CN model further identified RyR2 subdomains characterized by different spark probabilities, as observed in the 3D spark model. Secondary subdomains with lower spark probability were found in clusters containing two groups of channels separated by central narrow regions ∼ 2 – 3 channels in width. This lends meaning to the eigenvectors of the model, which define the membership of the RyR2s to each functional subdomain. Interestingly, ${\underline{v}}_{1}$ is a known measure of vertex centrality [43], which means that the proportion of all possible walks of length *k* beginning at vertex *j* is ${\left({\underline{v}}_{1}\right)}_{j}/({\underline{u}}^{T}{\underline{v}}_{1})$ when *k* is sufficiently large. This implies that the elements of ${\underline{v}}_{1}$ indicate the relative number of lattice walks beginning at each channel. Our results suggest that this is approximately true for clusters characterized by the dominant eigenmode, as channels with greater values of ${\left({\underline{v}}_{1}\right)}_{j}$ had higher spark probability. Because we consider the transient behavior of channel gating during a fixed time window, the assumption that *k* is large (i.e. *t* is large) may not hold, thus explaining why a subdominant eigenmode was observed for some clusters.

Our results indicate that the system is near the threshold under normal conditions, as the ratio *δ*/*β* is close to the threshold *λ*_1_. Therefore, small changes to *β* can greatly change the qualitative behavior of the system. Using a simple Ca^2+^ diffusion model, we determined that spark probability is sensitive to changes in biophysical parameters.

RyR2 open probability is modulated by a variety of factors including phosphorylation [32, 33], JSR Ca^2+^ concentration [34], oxidative stress [35], and genetic mutations [36, 37]. Most of these increase the opening rate of the channel and cause elevated Ca^2+^ spark frequency. Our recent work [4] and others [30, 44] have shown that RyR2 regulation by JSR Ca^2+^ concentration is not necessary for spark termination. Rather, depletion of the JSR Ca^2+^ stores causes a sufficient decrease in unitary RyR2 current such that the channel openings are not sustained. This mechanism is supported in the present model as well, as shown by the sharp increase in *δ*/*β* as *i*_*RyR*_ is decreased (see Fig 3B), as it would be due to reduction of the Ca^2+^ concentration gradient from inside the JSR to the subspace when RyR2s open.

A recent imaging study by Asghari et al. [45] observed regulation of RyR2 cluster structure. The authors reported RyR2s clusters in dense side-by-side lattices, as assumed in the present study, as well as checkerboard-like arrangements with greater spacing of ∼ 37 nm compared to the baseline of 31 nm. Increasing channel spacing uniformly caused an increase in *δ*/*β* to 6.3 at 37 nm (see Fig 3D). Note that for any graph whose vertices have a maximum of *m* neighbors, *λ*_1_ < *m* [27]. Therefore *λ*_1_ < 4 for cluster lattices. This suggests that any cluster in the checkerboard arrangement would be unlikely to exhibit Ca^2+^ sparks in the absence of other changes. Interestingly, checkerboard spacing was observed upon channel phosphorylation or after decreasing the cytosolic Mg^2+^ concentration, both of which increase RyR2 open probability [33, 34]. Therefore the increase in inter-channel spacing may counteract the effects of these conditions.

We maintained focus on the relevance of cluster morphology to Ca^2+^ spark probability when a single RyR2 opens spontaneously. Ca^2+^ release can also be triggered following electrical excitation of the cell due to Ca^2+^ influx through apposing LCCs located on the transverse tubule. Note modeling studies suggest that coupling fidelity between LCCs and RyR2s is still strong despite low spark probability [4, 5, 13]. This is because although LCC mean open time is shorter (∼ 0.5 ms), unitary LCC current is approximately the same as the RyR2, there are usually several LCCs per RyR2 cluster (the ratio of RyR2s to LCCs is 4–10 [46]), and LCC openings are synchronized upon membrane depolarization to drive local buildup of Ca^2+^.

**Fig 7:**
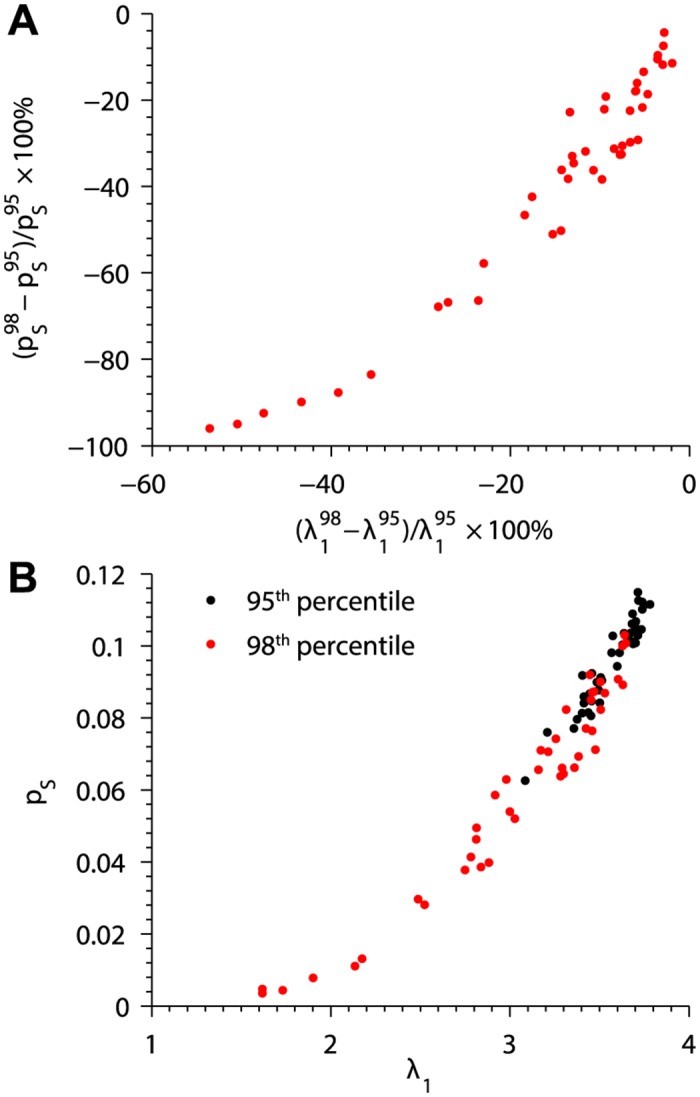
Dependence of *λ*_1_ and Ca^2+^ spark probability on RyR2 cluster image interpretation. (A) Percent changes in spark probability and *λ*_1_ for a collection of clusters reconstructed from STED images after raising the fluorescence intensity threshold from the 95^th^ percentile ($p_{S}^{95}$, $\lambda_{1}^{95}$) to the 98^th^ percentile ($p_{S}^{98}$, $\lambda_{1}^{98}$). (B) Relationship between *λ*_1_ and *p*_*S*_ when cluster structures are determined at the 95^th^ (*black*) and 98^th^ (*red*) percentiles.

The study of sub-cellular structure using super-resolution techniques requires careful interpretation of the raw image data. In this study, we generated RyR2 cluster lattices based on fluorescence intensity using a uniform thresholding algorithm. Intensities at or above the 95^th^ percentile were interpreted to represent the RyR2 positions over the entire image. To assess uncertainty in the results with respect to our choice of threshold, we analyzed a single set of STED images using both the 95^th^ and 98^th^ percentile thresholds. At the higher threshold, more of the fluorescence signal is filtered out and thus the clusters contain fewer RyR2s. This resulted in lower values of *λ*_1_ and decreased *p*_*S*_ (Fig 7A). The large differences in spark probability after using the higher threshold highlight the sensitivity of the model to the image processing methods. Nevertheless, there was still a strong correlation between *p*_*S*_ and *λ*_1_ when using the higher threshold (Fig 7B). Consequently quantitative prediction of spark probability applying *λ*_1_ requires consistent interpretation of super-resolution imaging data and in addition benefits from an incremental alteration of image analysis parameters if possible.

We did not consider weaker interactions between RyR2s such as those between diagonally-adjacent neighbors. This results in an underestimation of the open probabilities. We also did not consider clusters with heterogeneous inter-channel spacing as observed in Asghari et al. [45]. We also only considered single connected clusters containing no gaps that divide the cluster into separate subclusters. We assumed that the Ca^2+^ concentration gradient surrounding an open RyR2 declines sufficiently rapidly such that a negligible Ca^2+^ concentration is sensed in nearby subclusters, and therefore spark initiation occurs independently. These limitations could be overcome by using a distance matrix or diffusion model as in [47] to compute inter-RyR2 Ca^2+^ coupling. In addition, the LCN model is known to deviate most from the exact model near the stability threshold (*δ*/*β* ≈ *λ*_1_) [48]. Note it has been shown that the solution of the mean-field CN model is an upper-bound on the true probabilities [49], and although higher-order approximations do exist [42] we chose the first-order mean-field approximation for its simplicity and analytical tractability.

The CN model may also be applied to similar biological systems. It may be adapted to study Ca^2+^ release triggered by an LCC. The spark probability would be related to the coupling fidelity between LCCs and RyR2s. This model could be used to analyze the arrangement of LCCs as such experimental data become available. It may also be applied to future imaging studies to compare RyR2 cluster morphology to, for example, identify interspecies variability or remodeling in heart disease. For example, reduced RyR2 cluster sizes and fragmented JSR morphology have been observed in mouse models of catecholaminergic polymorphic ventricular tachycardia [50]. Inositol trisphosphate receptors (IP3Rs) located in the sarcoplasmic reticulum are known to aggregate into small clusters that exhibit similar release events known as Ca^2+^ “puffs,” and recent work has implicated cluster size in release extent [51] and trigger probability [52]. The present models could be used to compare IP3R cluster geometries like those reported in a recent study [53]. In skeletal muscle, Ca^2+^ release is coordinated mainly by physical LCC-RyR1 and RyR1-RyR1 interactions [54]. Imaging studies have observed that RyR1 clusters in slow-twitch muscle fibres were typically smaller and more fragmented than in fast-twitch muscle [1, 55]. The model presented here could be used to relate these observations to known differences in the Ca^2+^ release properties of these cell types.

More generally, the model could be adapted to complement super-resolution imaging studies of a wide range of receptors that form similar supramolecular clusters in other cell types [22, 23]. A general theoretical model has suggested that clusters of ligand-activated receptors behave cooperatively [56]. Examples from neurons include include synaptic microclusters of syntaxin 1 [57], acetylcholine receptor complexes at the neuromuscular postynapse [58], and rings of AMPA receptors found in spiral ganglion neurons [59]. Another example are immunoreceptors [60], which form clusters to amplify signal initiation and transduction, perhaps by decreasing the effective dissociation constants of ligands and downstream effectors [61]. Furthermore, Greenfield et al. employed super-resolution techniques to investigate the spatial organization of receptors involved in bacterial chemotaxis [62]. These receptors form clusters in the cell membrane and, similar to RyR2s, exhibit cooperative interactions that enhance sensitivity to low chemical signals.

This work presents a new perspective on cardiac calcium release and, more generally, highlights the relevance of subcellular variability in microdomains for the study of multi-scale biological systems.

## Methods and Models

### Ethics statement

All animal procedures were reviewed and approved by the Institutional Animal Care and Use Committee at University Medicine Göttingen Zentrale Tierexperimentelle Einrichtung (ZTE). Animal sacrifice was applied as described in Wagner et al. [24].

### Contact network model

Contact process models have been widely studied for their use in modeling disease and computer virus spread (see Keeling and Eams [41] for a review). In the present work, the CN model represents the RyR2 channel gating of a cluster of *n* channels. We will restrict ourselves to clusters that are connected, i.e. there are no separate islands of channels. The model is composed of a set of *n* random variables *X*_*i*_(*t*) = 1 if channel *i* is open at time *t* and 0 otherwise. If the channel is open, the probability that it closes within an infinitesimal time step *dt* is given by *δdt*, where *δ* = 0.5ms^−1^ is constant. If channel *i* is closed, it transitions into the open state in time *dt* with probability *βY*_*i*_(*t*)*dt*, where *Y*_*i*_(*t*) is the number of open adjacent channels. *β* is a constant given by *β* = *k* ^+^ *C* ^*η*^, where *k* ^+^ = 1.107 × 10^−4^ μM^−η^s^−1^ is the opening rate constant, *C* is the local elevation of Ca^2+^ concentration caused by an open neighbor, and *η* = 2.1 is the Hill coefficient for Ca^2+^ binding [4].

The adjacency matrix **A** is defined as an *n* × *n* matrix, where element (**A**)_*ij*_ = 1 if channels *i* and *j* are adjacent, and 0 otherwise. The number of open adjacent channels is then given by *Y*_*i*_(*t*) = ∑_*j*_(**A**)_*ij*_ *X*_*j*_(*t*). Let *p*_*i*_(*t*) = *P*(*X*_*i*_(*t*) = 1), the probability that channel *i* is open at time *t*, which obeys the equation [48]

$$\begin{array}{c} \frac{dp_{i}\left(t\right)}{dt}=\beta (1-X_{i}\left(t\right))\sum_{j=1}^{n}{\left(A\right)}_{ij}X_{j}\left(t\right)-\delta X_{i}\left(t\right). \end{array}$$ (4)

The entire system can be more compactly represented as the matrix equation

$$\begin{array}{c} \frac{d\underline{p}\left(t\right)}{dt}=(\beta \text{diag}\{\underline{u}-\underline{X}\left(t\right)\}A-\delta I)\underline{X}\left(t\right), \end{array}$$ (5)

where $\underline{p}(t)=[p_{1}(t),...,p_{n}(t)]$, $\underline{u}$ is the all-one-vector, $\underline{X}(t)=[X_{1}(t),...,X_{n}(t)]$, and **I** is the identity matrix. The system is therefore described by a set of *n* coupled stochastic differential equations, whose solution is analytically intractable. We simulated the CN model using the Gillespie algorithm [31]. Spark probability in the CN model was estimated by running an ensemble of 10,000 simulations per data point.

### Ca^2+^ diffusion model

Here we incorporate a simple model of Ca^2+^ diffusion that relate the CN model to the Ca^2+^-based communication between RyR2s. We use the steady-state diffusion equation for a continuous point source in a semi-infinite volume to obtain the Ca^2+^ concentration sensed by a RyR2 neighboring a single open channel [63]

$$\begin{array}{c} C=\frac{i_{RyR}}{2\pi zFd_{C}r}, \end{array}$$ (6)

where *i*_*RyR*_ = 0.15 pA is the unitary current of a single channel, *z* = 2 is the valency of Ca^2+^, F is Faraday’s constant, *d*_*C*_ is the effective diffusion coefficient of Ca^2+^ in the release site subspace, and *r* = 31 nm is the distance between the open channel pore and neighboring Ca^2+^ binding site.

The diffusion coefficient for Ca^2+^ in the subspace is unknown, though estimates for *d*_*C*_ in the cytosol range from 100 to 600 *μ*m^2^s^-1^ [64]. Ca^2+^ buffering molecules, electrostatic interactions with the membrane, and tortuosity imposed by the large RyR2 channels can affect the motion Ca^2+^ ions [65]. In light of these factors, the value of *d*_*C*_ was adjusted from 250 to 146 *μ*m^2^s^-1^ to obtain the nominal value of *β* = 0.115 that yields accurate spark probabilities (see Results).

### Linear mean-field contact network model

A common approach is to derive a mean-field approximation of the first moment of *X*_*i*_(*t*) by assuming that the higher moments are equal to 0 [48]. This yields a set of non-linear ordinary differential equations

$$\begin{array}{c} \frac{d\underline{p}\left(t\right)}{dt}=(\beta \text{diag}\{\underline{u}-\underline{p}\left(t\right)\}A-\delta I)\underline{p}\left(t\right), \end{array}$$ (7)

where $\underline{p}(t)$ is now the vector of mean-field open probabilities. This non-linear system is difficult to analyze analytically [48]. We further simplify the model by linearizing the equations about $\underline{p}=0$ [28]

$$\begin{array}{c} \frac{d\underline{p}\left(t\right)}{dt}=(\beta A-\delta I)\underline{p}\left(t\right). \end{array}$$ (8)

We refer to this as the linearized mean-field CN (LCN) model, which is amenable to the tools of linear systems theory. Note that the system is stable if and only if the maximum (dominant) eigenvalue of *β* **A** − *δ* **I**, given by *βλ*_1_−*δ*, is less than 0, or

$$\begin{array}{c} \lambda_{1}<\frac{\delta }{\beta }, \end{array}$$ (9)

where *λ*_1_ is the maximum (dominant) eigenvalue of **A**. Therefore, if *λ*_1_ < *δ*/*β*, the open probabilities in the LCN decay to 0. Otherwise, $\underline{p}(t)$ is unbounded as *t* → ∞. While physically meaningless, this result implies that the open probabilities increase when most channels are closed, or $\underline{p}(t)\approx 0$ (near the origin of linearization).

The eigendecomposition of **A** is given by

$$\begin{array}{c} A=VDV^{T}, \end{array}$$ (10)

where **V** is the modal matrix with columns formed by the orthonormal eigenvectors $\left\{{\underline{v}}_{1},...,{\underline{v}}_{n}\right\}$ of **A**, and **D** is a diagonal matrix of the eigenvalues {*λ*_1_, …, *λ*_*n*_} in descending order. Note that **A** is symmetric and therefore **V** ^−1^ = **V** ^*T*^. Combining Eqs (8) and (10) gives

$$\begin{array}{c} \frac{d\underline{p}\left(t\right)}{dt}=V\left(\beta D-\delta I\right)V^{T}\underline{p}\left(t\right), \end{array}$$ (11)

which can be rewritten as the summation

$$\begin{array}{c} \frac{d\underline{p}\left(t\right)}{dt}=\sum_{i=1}^{n}\left(\beta \lambda_{i}-\delta \right){\underline{v}}_{i}{\underline{v}}_{i}^{T}\underline{p}\left(t\right). \end{array}$$ (12)

The solution of this system is given by

$$\begin{array}{c} \underline{p}\left(t\right)=\sum_{i=1}^{n}e^{(\beta \lambda_{i}-\delta )t}{\underline{v}}_{i}{\underline{v}}_{i}^{T}\underline{p}\left(0\right). \end{array}$$ (13)

We refer to the eigenmodes as the eigenvalue-eigenvector pairs $\lambda_{i}-{\underline{v}}_{i}$. Note that $\underline{p}(t)$ is essentially a sum of the eigenmodes. If the initial probability distribution $\underline{p}(0)=\alpha {\underline{v}}_{i}$ for some constant *α*, then $\underline{p}(t)\propto {\underline{v}}_{i}$ for all *t*. In other words, the trajectory of the system will be entirely characterized by the *i* ^th^ eigenmode. In general, the contribution of the *i* ^th^ eigenmode is determined by the weight ${\underline{v}}_{i}^{T}\underline{p}(0)$ and a time-dependent exponential factor with time constant 1/(*βλ*_*i*_ − *δ*).

We define $\text{E}\left[{\underline{n}}_{O}(t)\right]$ as the vector whose elements ${\left(\text{E}\left[{\underline{n}}_{O}(t)\right]\right)}_{i}$ give the expected number of open channels at time *t* given that channel *i* is open initially. This is computed by taking the sum of the elements of $\underline{p}(t)$ in Eq (13)

$$\begin{array}{c} E[{\underline{n}}_{O}\left(t\right)]=\sum_{i=1}^{n}e^{(\beta \lambda_{i}-\delta )t}\left({\underline{u}}^{T}{\underline{v}}_{i}\right){\underline{v}}_{i}. \end{array}$$ (14)

We assume that in a resting RyR2 cluster, every channel experiences the same Ca^2+^ concentration and therefore is equally likely to initiate a spark. The expected total number of open channels when the first open channel is chosen randomly can be computed by setting $\underline{p}(0)$ to the uniform distribution and again summing over all elements of $\underline{p}(t)$

$$\begin{array}{c} E[N_{O}\left(t\right)]=\frac{1}{n}\sum_{i=1}^{n}e^{(\beta \lambda_{i}-\delta )t}{\left({\underline{u}}^{T}{\underline{v}}_{i}\right)}^{2}. \end{array}$$ (15)

## Supporting Information

S1 Dataset RyR2 cluster coordinate files. (ZIP)
